# Incipient schizophrenia: a case-based introduction to psychopathology and differential diagnosis - Clinical case

**DOI:** 10.1192/j.eurpsy.2024.109

**Published:** 2024-08-27

**Authors:** V. M. Maravic

**Affiliations:** ^1^ Institute of Mental Health; ^2^Faculty of Medicine, University of Belgrade, Belgrade, Serbia

## Abstract

**Abstract:**

The first psychotic episode is often a moment of utmost importance for the patients and their families, as well as for the treating professionals who need to address several dilemmas. Findings suggest that Self-Disorders (SD) specifically aggregate within schizophrenia spectrum disorders, pointing to the possibility that they could be a central phenotypic marker of schizophrenia spectrum disorders across the different severity of their clinical manifestations. We will present and discuss a case report involving a patient’s first admission, where diagnostic uncertainties exist. The goal is to enhance our comprehension of the significance of contemporary clinical and phenomenological psychopathology, with a specific focus on SD, in order to achieve a more accurate and precise assessment of incipient schizophrenia.

**Disclosure of Interest:**

None Declared

